# Supplementary figures and images for: RERconverge Expansion: Using Relative Evolutionary Rates to Study Complex Categorical Trait Evolution
Source: bioRxiv. 2023 Dec 7:2023.12.06.570425. Preprint. [Version 1] doi: 10.1101/2023.12.06.570425 (PMC10723433; doi:10.1101/2023.12.06.570425)

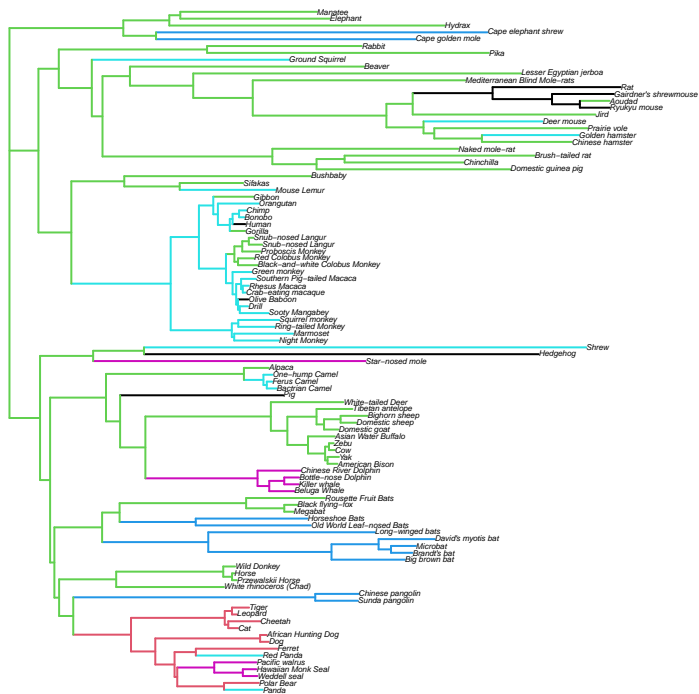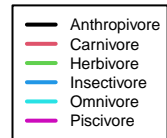

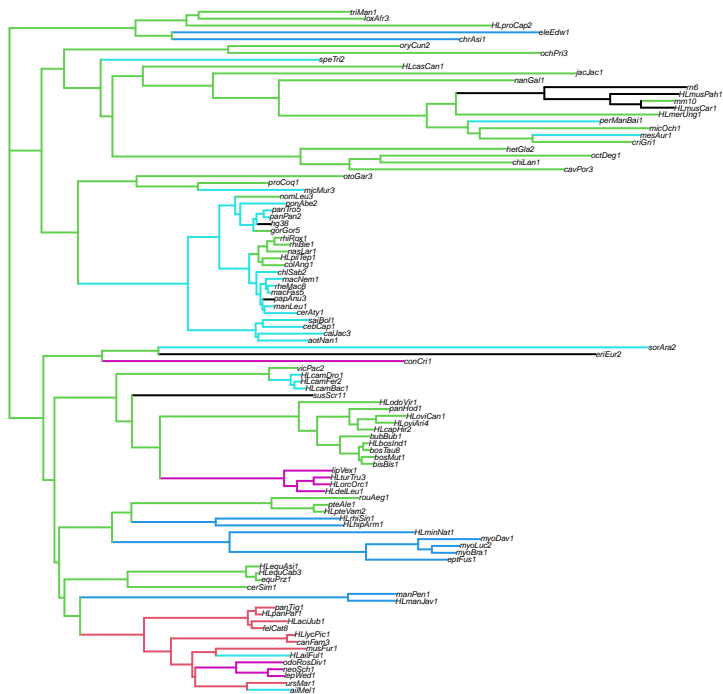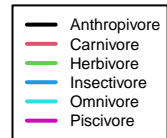

Supplement: Supplement 4 [file media-4.pdf]
