# Supplementary material for: RERconverge Expansion: Using Relative Evolutionary Rates to Study Complex Categorical Trait Evolution
Source: bioRxiv. 2023 Dec 7:2023.12.06.570425. Preprint. [Version 1] doi: 10.1101/2023.12.06.570425 (PMC10723433; doi:10.1101/2023.12.06.570425)
Supplement: 1 [file NIHPP2023.12.05.570141V1-supplement-1.pdf]

bioRxiv preprint doi: https://doi.org/10.1101/2023.12.06.570425; this version posted December 7, 2023. The copyright holder for this preprint (which was not certified by peer review) is the author/funder, who has granted bioRxiv a license to display the preprint in perpetuity. It is made available under aCC-BY 4.0 International license.

Supplementary Figures and Tables

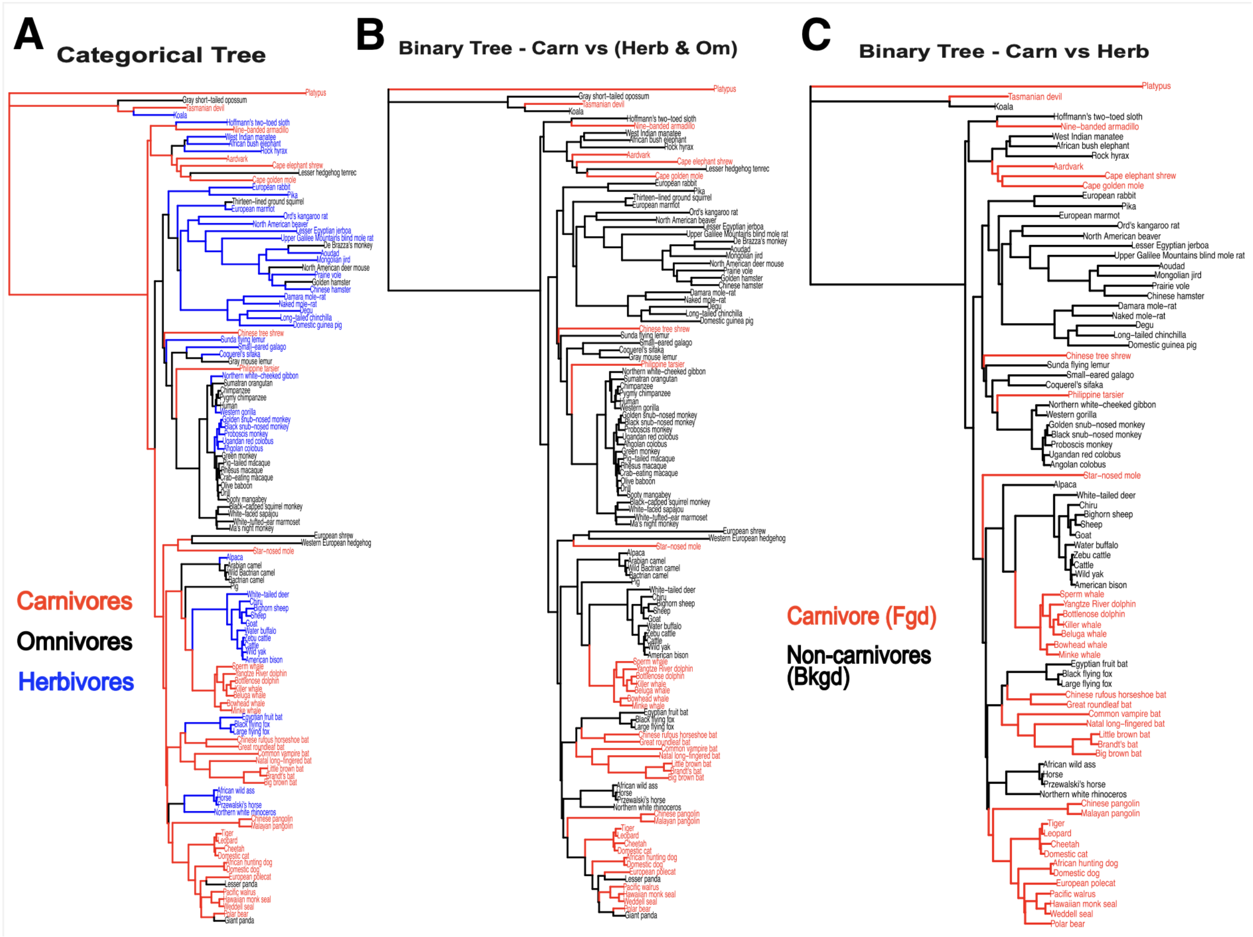

Figure S1. A) Categorical reconstruction using maximum likelihood applied to a continuous time markov model on the full phylogeny used in the analysis. B) Example of one of the binary reconstructions in which the foreground is carnivores and the background is herbivores and omnivores. Uses a maximum parsimony based approach. C) Example of one of the binary reconstructions in which the foreground is carnivore and the background is herbivores, with omnivores removed. Uses a maximum parsimony based approach.

bioRxiv preprint doi: https://doi.org/10.1101/2023.12.06.570425; this version posted December 7, 2023. The copyright holder for this preprint (which was not certified by peer review) is the author/funder, who has granted bioRxiv a license to display the preprint in perpetuity. It is made available under aCC-BY 4.0 International license.

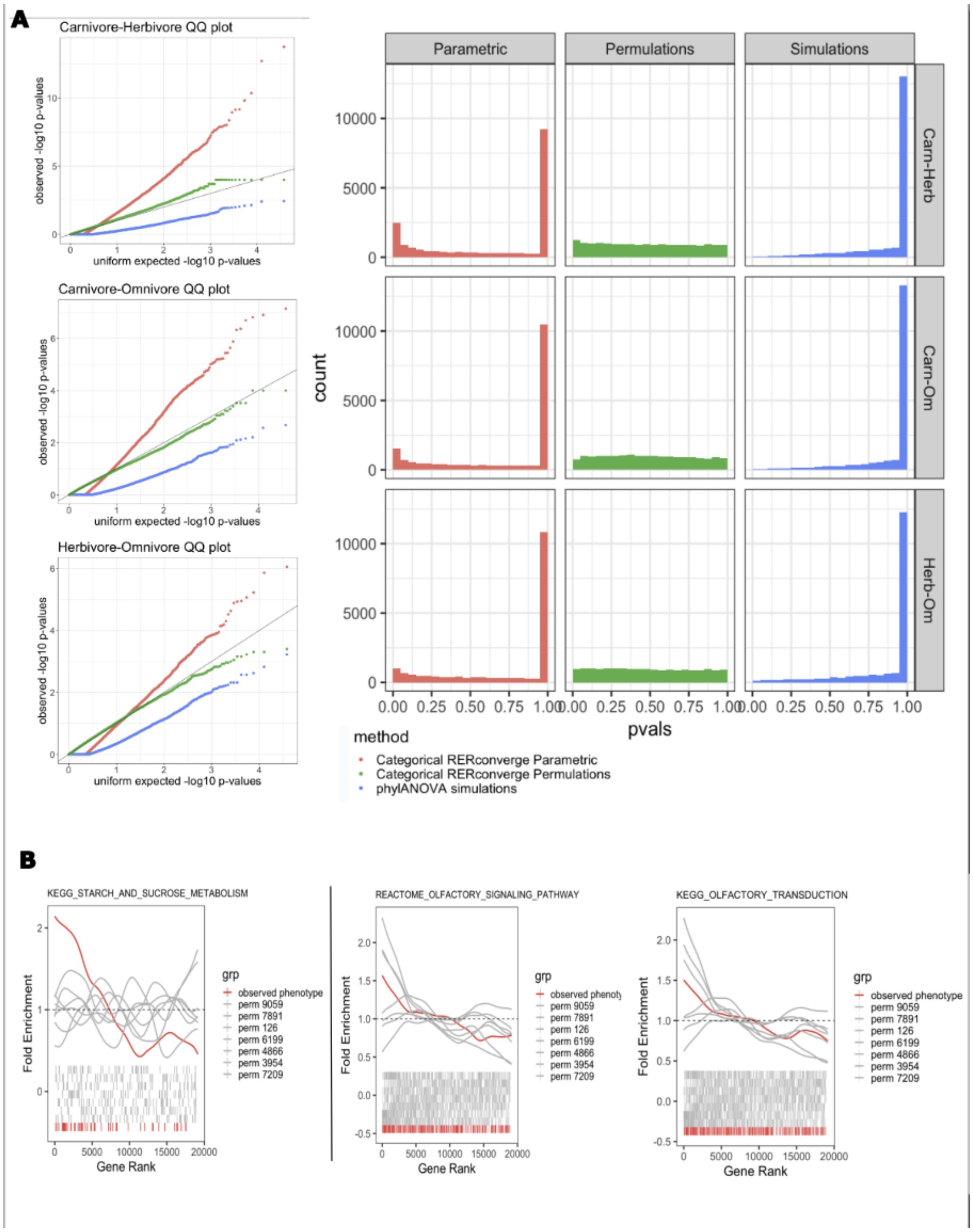

Figure S2. A) Quantile quantile plots and histograms of the, categorical RERconverge raw (parametric) p-values (red) and permulation p-values (green) and the phylanova simulation p-values (blue). B) Fold enrichment and barcode plots showing the enrichment of genes in the kegg starch and sucrose metabolism pathway (left) and two olfactory pathways (right). Red indicates the results for the observed phenotype, gray indicates the results for a random selection of seven (out of the 10,000) permulated phenotypes.

bioRxiv preprint doi: https://doi.org/10.1101/2023.12.06.570425; this version posted December 7, 2023. The copyright holder for this preprint (which was not certified by peer review) is the author/funder, who has granted bioRxiv a license to display the preprint in perpetuity. It is made available under aCC-BY 4.0 International license.

	Mean	SD	No Outlier Mean	No Outlier SD
Two Category	7.1987	0.428	7.198	0.428
Three Category	9.1044	0.951	9.1044	0.951
Four Category	38.591	34.49	32.614	18.64
Five Category	2358.0	5049.	302.38	214.1
Six Category	4933.0	NA	4933.0	NA

Table S1. Average time of all phenotype sets for each category number.

Relaxation (%)	comparison	Mean Difference (%)	Sd Difference (%)	Outlier Mean Difference (%)	Outlier Sd Difference (%)
Single	Single	Single	Single	Single	Single
20	3v2	2.510765	3.333216	2.510765	3.333216
20	4v3	32.56813	33.64818	32.56813	33.64818
20	5v4	155.0417	196.6984	355.9884	661.9572
20	6v5	320.1345	385.5756	320.1345	385.5756
10	3v2	23.12227	13.83585	23.12227	13.83585
10	4v3	284.4025	144.2443	376.9865	395.6866
10	5v4	1501.997	762.154	4703.116	6873.465
10	6v5	1715.987	2268.198	1715.987	2268.198
5	3v2	48.57902	27.11924	48.57902	27.11924
5	4v3	1195.116	835.5254	1195.116	835.5254
5	5v4	NaN	NA	NaN	NA
5	6v5	NaN	NA	NaN	NA
0	3v2	51.14266	30.8599	51.14266	30.8599
0	4v3	1591.495	1627.517	1591.495	1627.517
0	5v4	NaN	NA	NaN	NA
0	6v5	NaN	NA	NaN	NA

bioRxiv preprint doi: https://doi.org/10.1101/2023.12.06.570425; this version posted December 7, 2023. The copyright holder for this preprint (which was not certified by peer review) is the author/funder, who has granted bioRxiv a license to display the preprint in perpetuity. It is made available under aCC-BY 4.0 International license.

Double	Double	Double	Double	Double	Double
20	4v2	32.80652	NA	32.80652	NA
20	5v3	163.9363	88.87492	163.9363	88.87492
20	6v4	1028.2	NA	1028.2	NA
10	4v2	481.8241	NA	481.8241	NA
10	5v3	5014.968	3484.625	5014.968	3484.625
10	6v4	32220.14	NA	32220.14	NA
5	4v2	1947.827	NA	1947.827	NA
5	5v3	NaN	NA	NaN	NA
5	6v4	NaN	NA	NaN	NA
0	4v2	1173.703	NA	1173.703	NA
0	5v3	NaN	NA	NaN	NA
0	6v4	NaN	NA	NaN	NA

Table S2. Percent speed increases for reducing the number of categories in the analysis, at each relaxation level.

	Mean_0	Sd_0	Mean_5	Sd_5	%Dif_5	Mean_10	Sd_10	%Dif_10	Mean_20	Sd_20	%Dif_20
twoCategory	8.11	0.543	8.007	0.549	1.370	7.198	0.428	12.76	8.243	0.4890	-1.53157
threeCategory	14.35389	4.1969	13.883	5.0644	3.3853	9.1044	0.951	57.657	8.8042	0.5050	63.033
fourCategory	243.393	112.52	205.41	162.61	18.486	32.614	18.64	646.26	11.6372	2.3934	1991.49
fiveCategory	4320+	NA	4320+	NA	NA	302.38	214.1	NA	28.408	23.735	NA
sixCategory	4320+	NA	4320+	NA	NA	4933.0	NA	NA	119.679	NA	NA
twoCategoryAll	8.11748	0.543	8.0077	0.5498	1.3704	7.1987	0.428	12.762	8.2437	0.4890	-1.5315
threeCategoryAll	14.3538	4.1969	13.883	5.0644	3.3853	9.1044	0.951	57.657	8.8042	0.5050	63.033
fourCategoryAll	276.698	196.8	205.41	162.61	34.70	38.591	34.49	616.98	11.6372	2.3934	2277.6
fiveCategoryAll	4320+	NA	4320+	NA	NA	2358.0	5049.	NA	61.5213	96.201	NA
sixCategoryAll	4320+	NA	4320+	NA	NA	4933.0	NA	NA	119.679	NA	NA

bioRxiv preprint doi: https://doi.org/10.1101/2023.12.06.570425; this version posted December 7, 2023. The copyright holder for this preprint (which was not certified by peer review) is the author/funder, who has granted bioRxiv a license to display the preprint in perpetuity. It is made available under aCC-BY 4.0 International license.

Table S3. Table of the time average and standard deviations of time required to complete a phenotype set with a given number of categories, at each relaxation level tested. %Dif column represents the percentage speed increase of the relaxation level over 0% relaxation. As times for 0% and 5% relaxation for could not be completed, times were very conservatively estimated as the minimum time required to reach the run time produced (see methods), and no %Dif could be created.

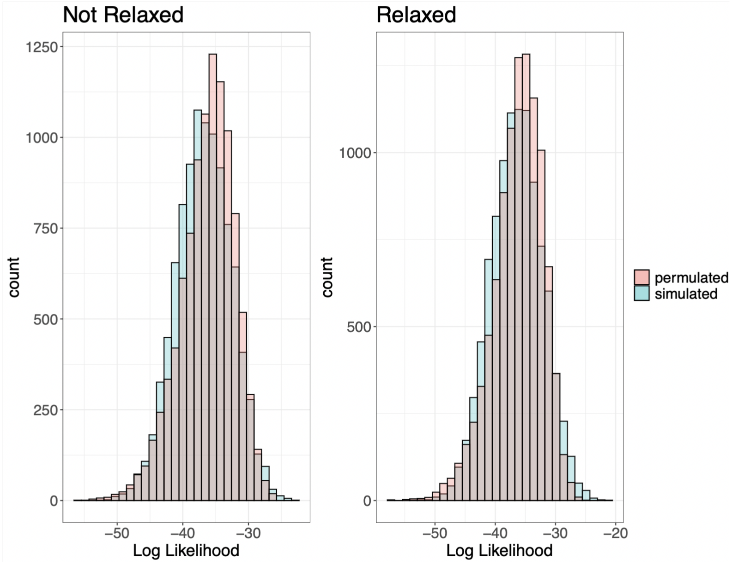

Figure S3. Histograms of the log likelihoods of the 10,000 simulated trees (blue) compared to the log likelihoods of the finished permulated trees (pink) for permulations without relaxation (left) and permulations with relaxation (right).

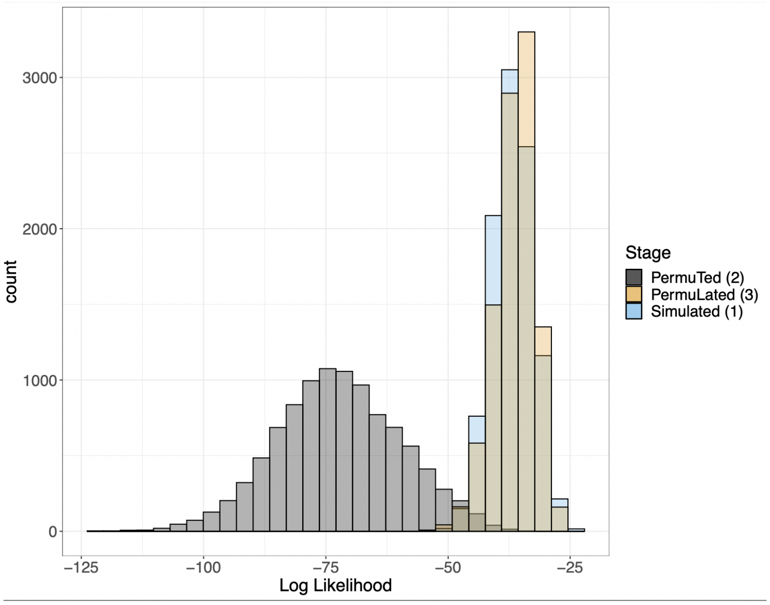

bioRxiv preprint doi: https://doi.org/10.1101/2023.12.06.570425; this version posted December 7, 2023. The copyright holder for this preprint (which was not certified by peer review) is the author/funder, who has granted bioRxiv a license to display the preprint in perpetuity. It is made available under aCC-BY 4.0 International license.

Figure S4. In blue are the log likelihoods of the 10,000 simulated trees (after step 1), in gray are the log likelihoods of the 10,000 permuted trees (after step 2), and in tan are the log likelihoods of the 10,000 finished permulated trees (after step 3).

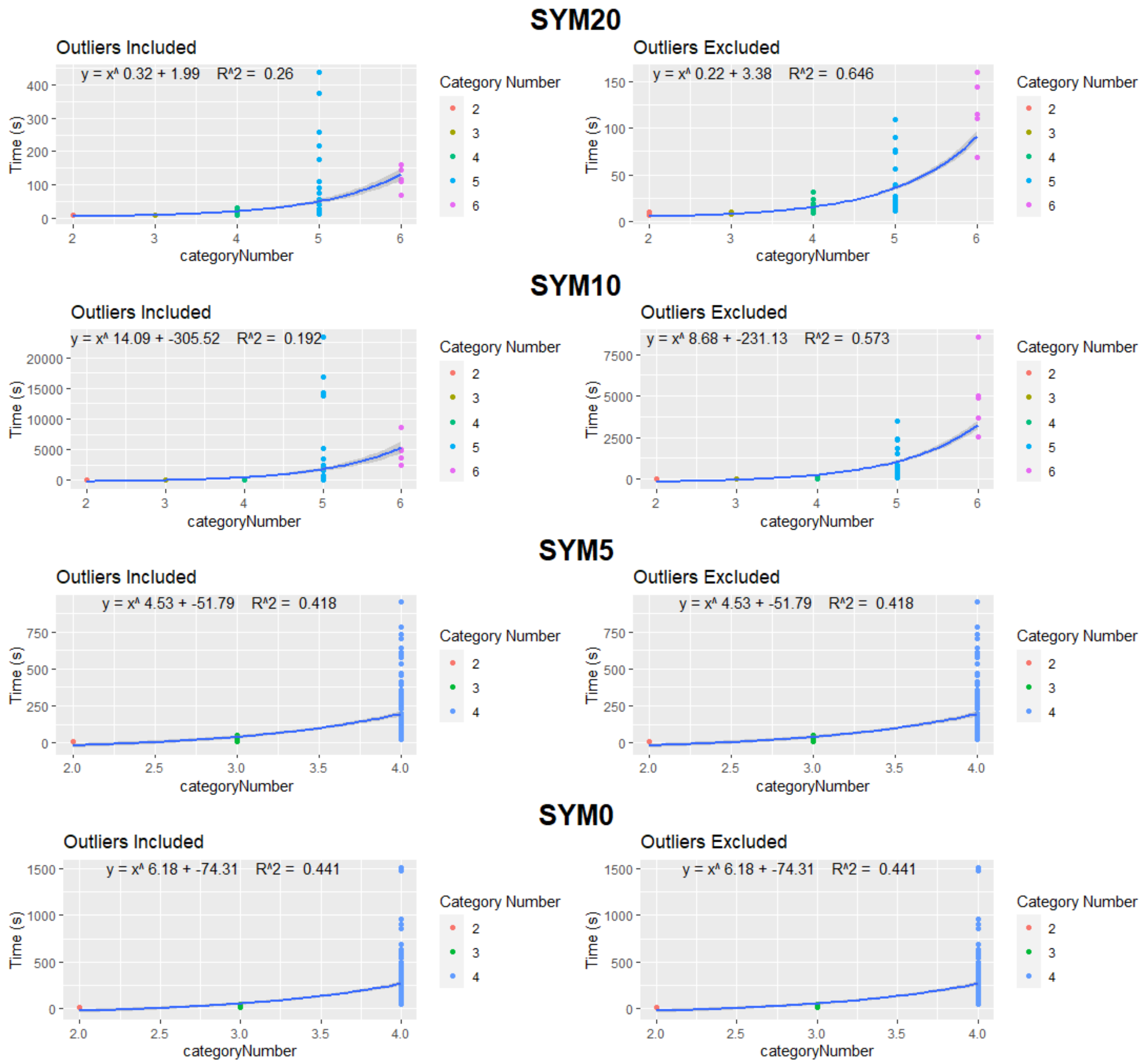

Fig S5. Graphs of the exponential effect of category number on permulations time. Note that the 5% and 0% relaxation graphs do not include 5 category or 6 category analyses.
